# An In-Depth Examination of Surgeon-Scientists’ NIH-Funded Areas of Research

**DOI:** 10.26502/jsr.10020254

**Published:** 2022-09-28

**Authors:** Lindsay A Demblowski, Andrew M Blakely, Martha A Zeiger

**Affiliations:** 1 Office of Surgeon Scientists Programs, Center for Cancer Research, National Cancer Institute, National Institutes of Health, Bethesda, MD, USA; 2 Surgical Oncology Program, National Cancer Institute, National Institutes of Health, Bethesda, MD, USA

**Keywords:** Academic surgery, Institutes or Centers, RCDC, Research focus

## Abstract

**Background:**

Few studies have examined which National Institutes of Health (NIH) Institutes or Centers (ICs) provide most of the funding to surgeons, nor examined the specifics of their research focus areas. A better understanding of both the goals of ICs and research focus areas for surgeons may facilitate further alignment of the two.

**Methods:**

A previously created database of NIH-funded surgeons was queried. To understand trends in funding, total grant cost was calculated for each IC in 2010 and 2020, and distribution of IC funds to each principal investigator (PI) category (surgeons, other physicians, and PhDs without a medical degree) was compared. Finally, total cost for Research Condition and Disease Categorization (RCDC) areas funded to surgeons compared to all of NIH was calculated. Statistical analyses were performed; a two-tailed *p* value of < 0.05 was considered significant.

**Results:**

The National Cancer Institute (NCI) awarded the largest percentage of all 2020 surgeon funding, 34.3% ($298.9M). Compared to the other ICs, surgeons held the largest percentage of the National Eye Institute’s (NEI) total funding in 2010 and 2020 at 8.7% and 9.0%, respectively. The RCDC super category comprising the most funding for surgeons was health disparities with 14.5% of all surgeon funding, followed by neurology (13.8%) and cancer (11.4%). Surgeons were awarded 10.8% of NIH’s transplant-related research, 7.0% of ophthalmology-related research, and 3.4% of cancer-related research in 2020.

**Conclusions:**

Our study shows surgeons have positioned themselves to examine new and myriad research topics while maintaining a focus on health disparities and cancer-related research.

## Introduction

The status of surgeons funded by the National Institutes of Health (NIH) has been reported upon over the last decade through a myriad of studies. Our recent study identified NIH grants awarded to surgeons, non-surgeon physicians, and PhDs without a medical degree in June of 2010 and compared workforce and surgeon-lead grant specifics, such as total costs and research type (basic science, clinical outcomes, or clinical trial), to June of 2020. We found that 1) the number of surgeon-scientists funded by the NIH has significantly increased from 2010 to 2020 compared to both non-surgeon physician-scientists and the surgeon workforce and, 2) surgeons have maintained a significant portion of their portfolio, more than 70%, in basic science research [1]. However, current literature has not examined either which NIH Institutes or Centers (ICs) provide the majority of NIH funding to surgeons, or the specifics of their research focus areas. Relevant to this, each IC has its own strategic plan aimed at aligning the mission and goals of the institute or center with its scientific priorities [2]. Therefore, a better understanding of both the goals of ICs and research focus areas for surgeons may facilitate further alignment of the two [3]. By way of example, a recent study by Dowd *et al.* found that the National Institute of General Medical Science (NIGMS) provided the largest funding to trauma research. The authors concluded that an established and well-funded institute offers more resources that could be utilized by PIs pursuing trauma research compared to starting a new, smaller institute solely focused in this area [4]. Furthermore, implemented in 2008 at the request of Congress to address a more transparent method of reporting the relationship between NIH’s research portfolio and public health needs, Research Condition and Disease Categorization (RCDC) was created. The RCDC categories include 292 condition, disease, or research areas that are linked to NIH grants [5,6]. Several studies have utilized RCDC information to further understand specific research topics, such as data science funded by the National Heart, Lung, and Blood Institute (NHLBI), trauma research, and research advocacy for schizophrenia, [4,7,8] but no study has used RCDC terms to examine the research of a specific group of NIH grant holders, in this case, surgeons. In our study, we sought to identify which ICs fund surgeon-scientists as well as which research areas surgeons focus on by utilizing RCDC categories. To accomplish this, we queried a previously created comprehensive database of NIH funding for surgeons [1]. Herein, we report on IC funding for surgeon-scientists, including total costs awarded to surgeons by each IC in June of 2010 compared to 2020. We also compare the overall proportion of IC funding awarded to surgeons, other physicians, and PhD principal investigators (PIs) in both years. Lastly, we report on RCDC super categories associated with grants held by surgeons in 2020 compared to all of NIH, as well as the distribution of R01 grant research type of each super category.

## Methods

### 2010 and 2020 Surgeon-scientist Databases

A previously created database [1] that included NIH-funded surgeons, other physicians (MD, MBBS, and/or DO), and PhDs without medical degrees, curated in the internal NIH data platform, iSearch *Grants* (NIH Office of Portfolio Analysis’s next-generation portfolio analysis platform; v2.4) [9], was queried for this analysis. Surgeons were identified by searching key terms within NIH biographical sketches and verified based upon credentials obtained from university or academic online profiles. Cardiothoracic, general (including all general surgery-derived subspecialties), neurological, obstetrics and gynecology, ophthalmic, orthopaedic, otolaryngology, plastic and reconstructive (including oral-maxillofacial), urological, and vascular surgeons were included in the database. R01 grants were categorized into one of three research types: basic science, clinical outcomes, or clinical trials, based on grant titles and specific aims. The following information was pulled from the database for all grants awarded to surgeons in June 2010 and June 2020: PI name and PI number, grant title, specific aims, activity code, RCDC, administrative IC, grant number, and total cost.

All research-focused grants active in June of 2010 and June of 2020 were included in the analysis. The following non-research grant activity codes were excluded: Research Construction Programs (C06), Resource Programs (G08, G12, and G20), Loan Repayment Programs (L30 and L40), General Clinical Research Centers Program (M01), Other Transactions (OT2), and Research-Related Programs (S10), as these grants are considered primarily programmatic and not reflective of PI research.

### NIH Institutes and Centers Analysis

The administrative IC is responsible for awarding grants in compliance with its current budget and funding strategies [10]. Total cost and the total number of grants awarded to surgeons from each IC was calculated and compared 2010 *vs.* 2020. In addition, the number of PIs funded by each IC for both 2010 and 2020 were used to calculate cost per PI for each IC in both years. The proportion of awarded costs to the PI categories (surgeons, other physicians, and PhDs) from each IC was also calculated and compared 2010 *vs.* 2020.

The following 10 institutes were included in this analysis as they represented the majority of funding (90.4%) awarded to surgeons in 2020: National Cancer Institute (NCI), National Eye Institute (NEI), National Heart, Lung, and Blood Institute (NHLBI), National Institute of Allergy and Infectious Diseases (NIAID), National Institute of Arthritis and Musculoskeletal and Skin Diseases (NIAMS), National Institute of Child Health and Human Development (NICHD), National Institute on Deafness and Other Communication Disorders (NIDCD), National Institute of Diabetes and Digestive Kidney Diseases (NIDDK), National Institute of General Medicine Sciences (NIGMS), and National Institute of Neurological Disorders and Stroke (NINDS) (Table 1).

Other ICs not included and that awarded surgeons’ funding in 2010 and 2020 included: FIC, NCATS (2020 only), NCCIH, NCRR (2010 only), NHGRI, NIA, NIAAA, NIBIB, NIDA, NIDCR, NIEHS, NIMH, NIMHD, NINR (2020 only), NLM, OD (2010 only) [2]. Funding from each represented < 2.0% of the respective IC total 2020 costs awarded to surgeons.

### Research Condition and Disease Categorization Analysis

RCDC terms linked to grants held by surgeons in June of 2020 were identified, and grants without an RCDC term were excluded from this analysis. Notably, grants are often associated with several RCDC terms that are not mutually exclusive. Therefore, a grant’s total cost is reported alongside every RCDC term linked to the grant, making total costs associated with RCDC terms significantly more than the actual total NIH funds available. This process is used by NIH when reporting yearly RCDC totals for NIH [6]. Furthermore, only terms that were included in the estimates of funding for 2020 for both NIH and surgeons were included in this analysis, resulting in 196 of the 292 RCDC terms related to surgical research. Due to the political impetus behind the use of RCDC terms in combination with the funding overlap and variability in reported totals, these terms only indicate relative research focus areas [5,6].

In order to create a manageable and more comprehensible list of research focus areas, we sorted the 196 RCDC terms into 24 super categories. These super categories included: Cancer, Cardiovascular, Digestive and Bariatric, Endocrine, Fetal, Genetics, Head and Neck, Health Disparities, Hepatic, Infectious Diseases, Musculoskeletal, Neurology, Obstetrics and Gynecology, Ophthalmology, Outcomes, Pediatrics, Psychology, Pulmonary, Radiology, Stem Cell Research, Substance Abuse, Technology, Transplant, and Urology. A complete list of the 24 super categories and RCDC terms from this analysis are detailed in Appendix A. We calculated total costs for each super category and compared surgeon costs to all of NIH as a percentage of total funds for 2020 [6]. We then determined the distribution of RCDC total costs associated with R01 grants within each super category stratified by research type: basic science, clinical outcomes, and clinical trials. The median percentage and range were calculated for each research type.

### Statistical Analysis

Data were expressed as IC total costs and treated as units of millions of dollars. Stratification by assessment year and awardee category (surgeons *vs.* non-surgeon) was performed. Bivariate chi-squared analyses were performed using total costs. Then, *z-*tests of funding proportions were performed, using the proportion from 2010 as the null hypothesis. The data were analyzed in two stages. The first analysis evaluated total funding from each IC awarded to surgeons compared to non-surgeons for 2010 *versus* 2020. The second analysis evaluated each individual IC in terms of percentage of overall IC funding that was awarded to surgeons in 2010 *versus* 2020. Finally, the corresponding RCDC super categories for surgeon funding were also provided in a descriptive fashion, analyses were limited to the top ten IC funding bodies listed above. Statistical analyses were performed using JMP, Version 14.0.0 (SAS Institute, Cary, NC). A two-tailed *p* value of < 0.05 was considered significant.

## Results

### IC funding to surgeon-scientists

#### Distribution of all surgeon funding 2010 vs. 2020

Per our prior study, in June of 2020, surgeons held $872.5M in NIH funding compared to $614.7M in June of 2010, 2.3% and 1.9% of total NIH funding, respectively [1]. According to the U.S. Bureau of Labor Statistics’ estimation of 1.18% inflation rate from June 2010 to June 2020, surgeons still significantly increased their total funding from 2010 ($725.3M) to 2020 ($872.5M) (*p*<0.05) [11].

**Figure 1:**
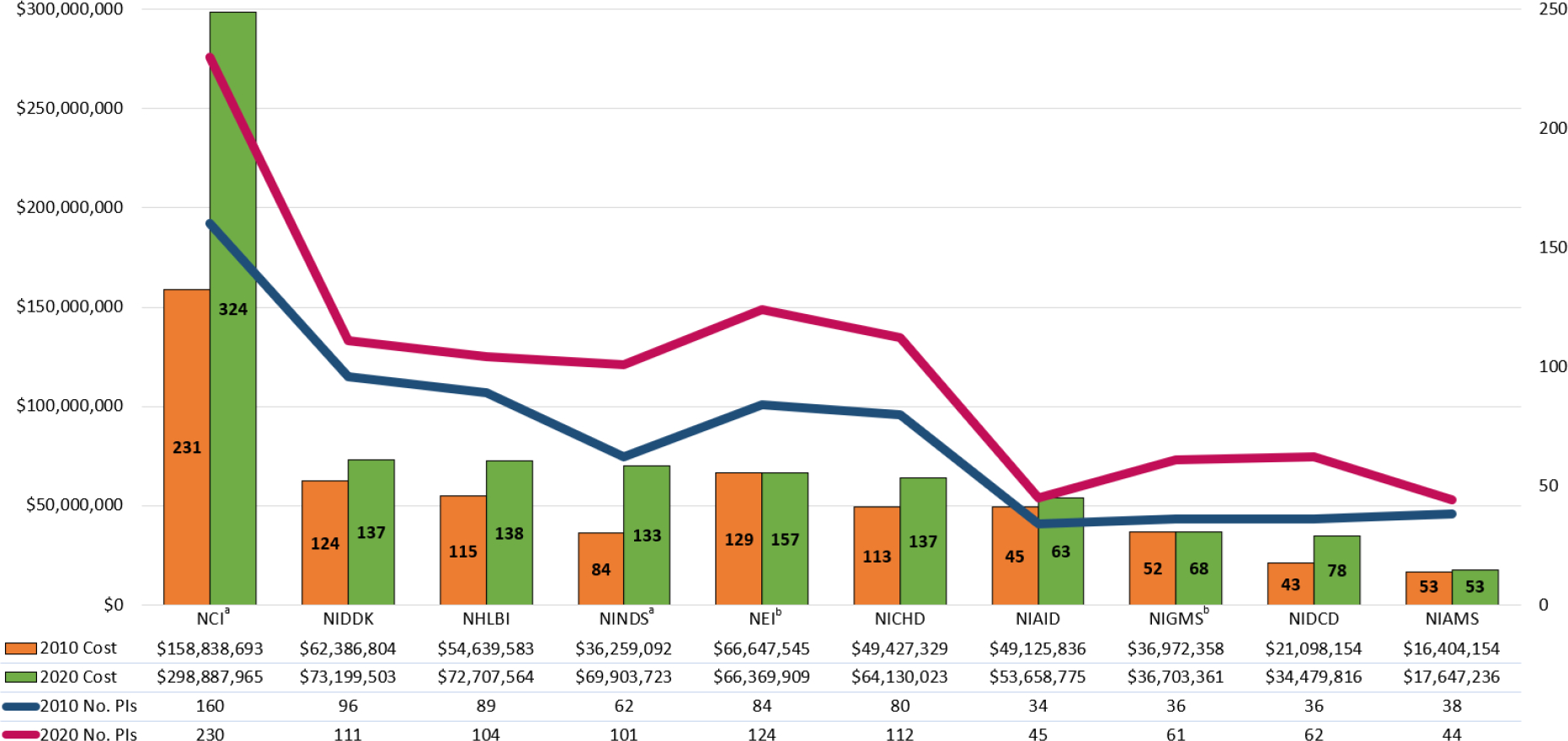
Number of Surgeon-Scientists and Total Funding by NIH IC 2010 *vs.* 2020. Includes the top ten ICs comprising 90% of funding awarded to surgeons in 2020. A significant ^a^ increase in total awarded grant costs as a proportion of total surgeon funding was observed for NCI and NINDS (*p*<0.05). NEI and NIGMS funding significantly ^b^ decreased in proportion of total surgeon funding from 2010 to 2020 (*p*<0.05), even though total amount of funding remained stable. The number of grants funded by each IC are listed within the bars for both years.

NCI awarded the largest percentage of all funding to surgeons, 34.3% ($298.9M) in 2020, followed by NIDDK, 8.4% ($73.2M), and NHLBI, 8.3% ($72.7M) (Figure 1). In comparison, in 2010, NCI awarded the largest percentage of surgeons’ total funding, 25.8% ($158.8M), followed by NEI, 10.8% ($66.6M), and NIDDK, 10.1% ($62.4M). When comparing IC funding from 2010 to 2020, a significant increase in total awarded grant costs as a proportion of total surgeon funding was observed for NCI (*p*<0.05) and NINDS (*p*<0.05). While NEI and NIGMS significantly decreased in proportion of total funding from 2010 to 2020 (*p*<0.05), the total amount of funding remained stable. No other IC observed a significant increase or decrease in proportion of total funding awarded to surgeons from 2010 to 2020.

During this time, the number of grants awarded to surgeons also increased from each IC. In 2010, NCI awarded 231 grants to surgeons and 324 in 2020 (Figure 1). A similar increase in both number of grants and total funding was observed for NIDDK (124, 137), NHLBI (115, 138), NINDS (84, 133), NICHD (113, 137), and NIDCD (43, 78). NEI and NIGMS observed an increase in the number of grants awarded to surgeons but did not a see an increase in the amount of funding. No IC awarded fewer grants to surgeons in 2020 compared to 2010.

National Institute of Health (NIH), Institutes or Centers (IC), National Cancer Institute (NCI), National Institute of Diabetes and Digestive Kidney Diseases (NIDDK), National Heart, Lung, and Blood Institute (NHLBI), National Institute of Neurological Disorders and Stroke (NINDS), National Eye Institute (NEI), National Institute of Child Health and Human Development (NICHD), National Institute of Allergy and Infectious Diseases (NIAID), National Institute of General Medicine Sciences (NIGMS), National Institute on Deafness and Other Communication Disorders (NIDCD), National Institute of Arthritis and Musculoskeletal and Skin Diseases (NIAMS)

Furthermore, the total number of surgeon PIs funded by each IC increased from 2010 to 2020 (Figure 1). While NCI funded 160 surgeons in 2010, 230 were funded in 2020. The NCI cost per PI also increased from $992,742 to $1,299,513, respectively (Appendix B). However, no other IC had a large increase in cost per PI. The largest cost per PI in 2010 was awarded to surgeons from NIAID, $1,444,878 (34 surgeons), which decreased to $1,192,417 (45 surgeons) in 2020. The largest drop in cost per PI compared to the other ICs was observed for NIGMS, which awarded grants to 36 surgeons in 2010 ($1,027,010 per PI) and 61 surgeons in 2020 ($601,694 per PI) while total cost essentially stayed the same for both years.

#### Surgeons compared to entire IC’s 2010 vs. 2020 funding

**Figure 2:**
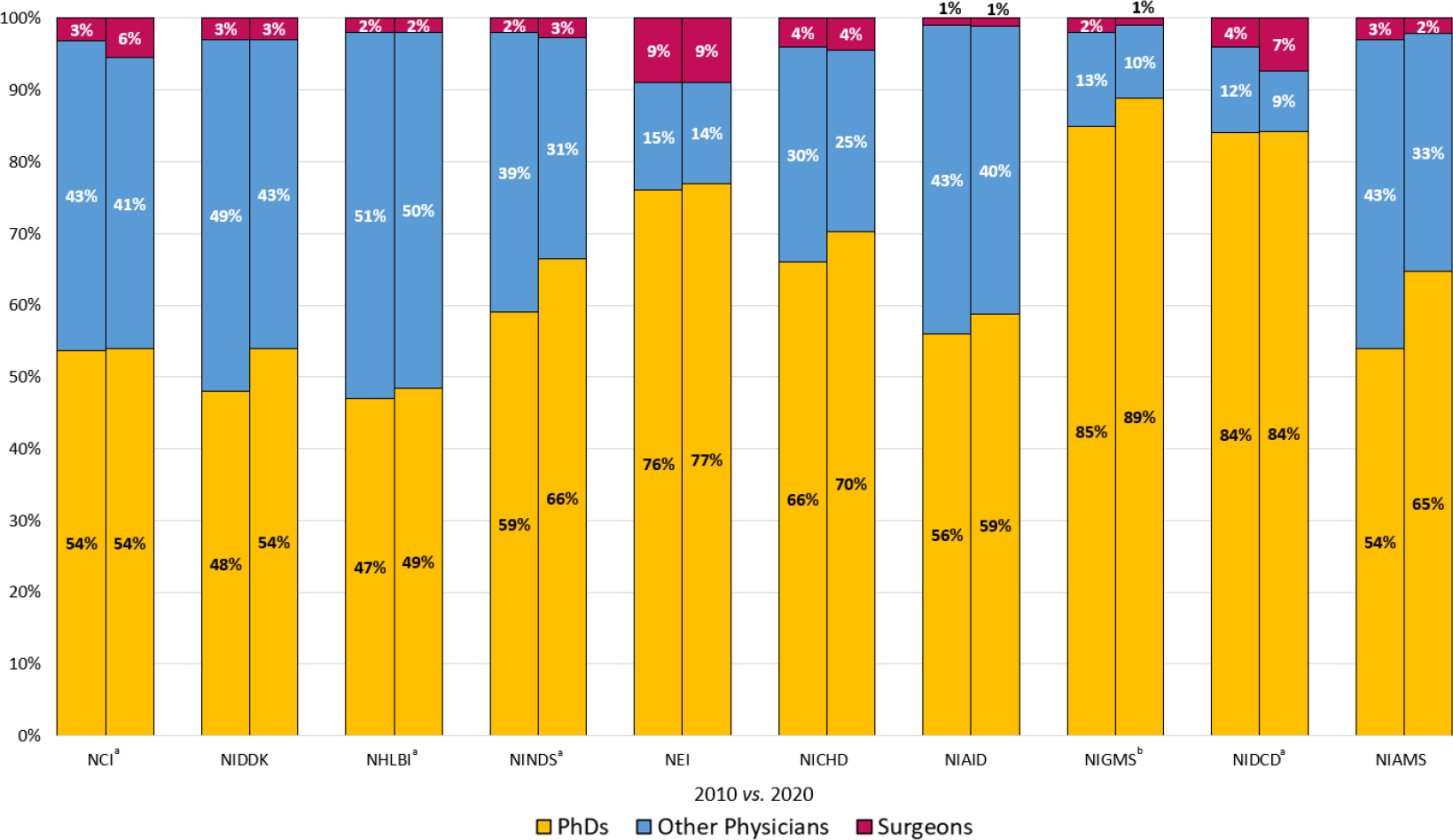
Percentage of Total NIH IC Funding Awarded to PI Categories 2010 *vs.* 2020 Includes the top ten ICs comprising 90% of funding awarded to surgeons in 2020. A significant ^a^ increase in proportion of total IC funding awarded to surgeons compared to all non-surgeons was observed for NCI, NHLBI, NINDS, and NIDCD (*p*<0.05). NIGMS funding awarded to surgeons significantly ^b^ decreased in proportion of total IC funding compared to all non-surgeons from 2010 to 2020 (*p*<0.05). *n.b.* Percentages are rounded to the nearest whole number.

IC funding to surgeons was then compared to the total funding from each IC for that year. Compared to the other ICs, surgeons held the largest percentage of NEI’s funding in both 2010 and 2020 at 8.7% and 9.0%, respectively (Figure 2). NIDCD significantly increased its funding to surgeons from 4.4% of its 2010 total costs to 7.3% in 2020 (*p*<0.05). A significant increase in proportion of funding awarded to surgeons compared to all non-surgeons from 2010 to 2020 was also observed for NCI, from 3.2% to 5.5%, NINDS, 1.9% to 2.7%, and NHLBI, 1.5% to 2.0% (*p*<0.05). NIGMS significantly decreased its funding awarded to surgeons, from 2.2% in 2010 to 1.0% in 2020 (*p*<0.05). Lastly, the proportion of IC funding awarded to surgeons remained stable for the remaining ICs.

National Institute of Health (NIH), Institutes or Centers (IC), National Cancer Institute (NCI), National Institute of Diabetes and Digestive Kidney Diseases (NIDDK), National Heart, Lung, and Blood Institute (NHLBI), National Institute of Neurological Disorders and Stroke (NINDS), National Eye Institute (NEI), National Institute of Child Health and Human Development (NICHD), National Institute of Allergy and Infectious Diseases (NIAID), National Institute of General Medicine Sciences (NIGMS), National Institute on Deafness and Other Communication Disorders (NIDCD), National Institute of Arthritis and Musculoskeletal and Skin Diseases (NIAMS)

### Research Conditions and Disease Categorization

The RCDC super category comprising the most funding awarded to surgeons only was health disparities, 14.5% of all surgeon funding, followed by neurology (13.8%) and cancer (11.4%) (Table 2). In terms of percentage of total funding to super categories from all of NIH, surgeons were awarded 6.6% of obstetrics and gynecology-related research, 4.7% of health disparities-related research, and 3.4% of cancer-related research. While neurology was the second most funded category for all surgeons, they only held 1.5% of all NIH neurology-related research. The Transplant super category comprised only 2.6% of surgeon funding, yet 10.8% of all NIH transplant-related funding was awarded to surgeons. Similarly, 7.0% of all ophthalmology-related funding was awarded to surgeons, but only comprised 2.0% of surgeons’ funding. NIH awarded more than 5% of all Urology, Head and Neck, and Fetal super category funding to surgeons, while surgeons held less than 2% of funding of their total RCDC funding to each of these categories.

Six hundred and twenty-seven R01 grants with associated RCDC terms were awarded to surgeons in 2020. The median percentage for each of the 24 super categories’ basic science grants was 71.8% of total costs, ranging from 49.8% to 85.3% (Table 3). The median percentage of clinical outcomes grants was 19.2% of total costs, ranging from 9.2% to 39.2%, and the median percentage of clinical trial grants was 8.7% of total costs, ranging from 0.0% to 16.1%. Musculoskeletal and psychology-related research awarded more than 80% of their funding to basic science, and only one category, fetal-related research, awarded less than 50% of its funding to basic science research.

## Discussion

Our study aimed to better understand the NIH-funded research areas surgeon-scientists focus on by analyzing both IC distribution of funding to surgeons and surgeons’ research topics. We found that from 2010 to 2020, NCI remained the largest funding IC for surgeons, with a significant 10-year increase in the proportion of overall funding awarded to surgeons. Additionally, NEI remained the IC awarding the largest percentage of its funding to surgeons from 2010 to 2020 (8.7% to 9.0%, respectively). By examining RCDC we also found that health disparities research comprised the majority of surgeons’ research focus, followed by neurology- and cancer-related research. Unexpectedly we also found that basic science comprised about 50–85% of each research topic’s total funding. Using NIH RePORTER, Smithson *et. al.* found NIGMS, NCI, NHLBI and NIDDK to be the ICs to most often fund surgical department applicants from 1998 to 2018 [12]. Similarly, Mesquita-Neto *et. al.* found NCI, NHLBI, NIDDK, NIAID, and NIGMS to be the NIH ICs to most frequently fund new awards to surgical departments from 2008 to 2018 [13]. These results mirror our study’s findings, namely, the top ten NIH ICs to fund surgeons in 2010 and 2020 were: NCI, NIDDK, NHLBI, NINDS, NEI, NICHD, NIAID, NIGMS, NIDCD, and NIAMS. However, our data differs from these prior studies in that iSearch *Grants* rather than NIH RePORTER was utilized to obtain the publicly available data [1]. Our study included all surgeons found within iSearch *Grants* after querying biographical sketches of the grant-holding PIs rather than depending on departmental filters in NIH RePORTER [1]. When filtering by department in NIH RePORTER, only those departments affiliated with US medical schools are captured, and organizations with different departmental structures are not included in the query. By way of example, institutions such as Massachusetts General Hospital are not included in the database when querying by department but, are included when searching by PI or grant number [3]. Furthermore, our study also includes a broad representation of grant types, including all grants actively awarded to surgeons in June of 2010 and June of 2020 as well as all surgical specialties. Other studies include, for example, new R-series grants [13] or R01 grants and career development awards only [12]. In order to examine surgeon research focus areas, we queried RCDC terms. It is important however to understand that this analysis has its limitations. For example, text data mining is used to match grants to research categories, resulting in only best estimates [6]. The method is susceptible to semantics, especially over time, such as the shift from the term ‘health inequalities’ to ‘health disparities’ [14]. Additionally, the categories are not all mutually exclusive, resulting in more than one hundred percent of all NIH funding, and therefore the number of surgeons and/or grants, to be included in the dataset [6]. Furthermore, multiple terms are associated with each grant, thereby increasing the broad representation of research focus areas rather than pinpointing a research focus area of a particular group, or in this case, surgical specialty. Finally, RCDC super categories have a variable number of terms, resulting in grant cost to exponentially increase as the number of related terms per grant increases. In summary, although aligning specific RCDC terms to grants results in defining trends in focus areas, the data are imperfect. Our results show health disparities-related research to be the largest RCDC super category funded to surgeons. This is no surprise in that Kneipp *et. al.* reported that the NIH made a significant investment in health disparities research through research center and training grant mechanisms between the years 2000 and 2016 [14]. Neurology-related research was the second largest RCDC super category for surgeons in 2020, as evidenced by the fact that neurological surgeons more than doubled their funding from 2010 to 2020 [1]. A similar pattern was observed for cancer-related research and transplant-related research; both surgical oncology and transplant surgeons had a large increase in the number of surgeons and total costs awarded from 2010 to 2020 [1], aligning surgical specialties and research focus areas. The 24 RCDC super categories span a wide array of research areas of focus, suggesting surgeon-scientists are key players in advancing novel research areas. Indeed, these include the genetic aspects of surgical diseases, stem cell research, as well as nanotechnology, highlighted by Dr. B Mark Evers in his 2015 Southern Surgical Association Presidential Address [15].

## Conclusions

Our study represents an in-depth look at which NIH ICs fund surgeons’ research as well as their areas of research focus. Importantly, we show key ICs have either significantly increased the proportion of funding to surgeons or at a minimum, maintained the percentage over the last decade. As NIH’s total funding grows, and therefore each IC total funding also increases, surgeons continue to successfully obtain funding. While surgeons’ research spans a broad representation of focus areas, a clear alignment between, funding ICs, and/or research focus area was observed in our study. Despite literature suggesting surgeon-scientists are threatened, our study shows surgeons have positioned themselves to be involved in new and myriad research topics while maintaining a focus on health disparities, neurology- and cancer-related research.

## Figures and Tables

**Table 1:** Abbreviations and Names of Top Ten NIH ICs Funding Surgeons

Abbreviation	NIH Institute or Center Name
NCI	National Cancer Institute
NEI	National Eye Institute
NHLBI	National Heart, Lung, and Blood Institute
NIAID	National Institute of Allergy and Infectious Diseases
NIAMS	National Institute of Arthritis and Musculoskeletal and Skin Disorders
NICHD	National Institute of Child Health and Human Development
NIDCD	National Institute on Deafness and Other Communication Disorders
NIDDK	National Institute of Diabetes and Digestive and Kidney Diseases
NIGMS	National Institute of General Medicine Sciences
NINDS	National Institute of Neurology Disorders and Stoke

**Table 2:** RCDC Super Category Percentage of Total Costs Awarded to Surgeons

RCDC Super Category	Percentage of Total Surgeon Funding (%)	Surgeon Percentage of Total NIH Funding (%)
Health Disparities	14.5	4.7
Neurology	13.8	1.5
Cancer	11.4	3.4
Technology	8.3	2.1
Genetics	6.9	1.8
Outcomes	6.6	1.8
Pediatrics	4.7	2.6
Infectious Diseases	4.4	0.9
Cardiovascular	3.8	2.6
Obstetrics and Gynecology	3.3	6.6
Psychology	3.3	0.8
Digestive and Bariatric	3.2	2.1
Transplant	2.6	10.8
Ophthalmology	2	7
Urology	1.7	5.5
Radiology	1.4	2.2
Stem Cell Research	1.3	2.4
Musculoskeletal	1.3	1.9
Head and Neck	1.2	5.6
Endocrine	1	3
Hepatic	1	3.1
Substance Abuse	0.9	0.6
Pulmonary	0.9	1.1
Fetal	0.6	5.4

Research Condition and Disease Categorization (RCDC), National Institutes of Health (NIH)

**Table 3:** Distribution of R01 Grant Type Total Costs by RCDC Super Category

*RCDC Super Category*	Basic Science (%)	Clinical Outcomes (%)	Clinical Trials (%)
Cancer	68.5	21.8	9.6
Cardiovascular	73.8	18.9	7.3
Digestive and Bariatric	66.4	21.2	12.4
Endocrine	62.3	21.8	15.9
Fetal	49.8	39.2	11
Genetics	74.1	16.9	9
Head and Neck	71.7	18.1	10.2
Health Disparities	72	18.8	9.2
Hepatic	54.1	30.2	15.7
Infectious Disease	77	13.7	9.3
Musculoskeletal	85.3	10.7	4
Neurology	76.5	17.9	5.6
Obstetrics and Gynecology	71.3	23	5.7
Ophthalmology	68	23.5	8.5
Outcomes	73.7	16.6	9.7
Pediatrics	70.8	25.3	4
Psychology	84.9	9.2	5.8
Pulmonary	64.3	19.6	16.1
Radiology	77.2	16.4	6.3
Stem Cell Research	74.9	19.5	5.6
Substance Abuse	65.5	34.5	0
Technology	74	17.6	8.4
Transplant	59.1	31.9	9
Urology	74.4	17.3	8.3

Research Condition and Disease Categorization (RCDC)

## Abbreviations

IC: Institute or Center
NIH: National Institutes of Health
PI: Principal Investigator
RCDC: Research Condition and Disease Categorization
